# Effect of Stomatal Morphology on Leaf Photosynthetic Induction Under Fluctuating Light in Rice

**DOI:** 10.3389/fpls.2021.754790

**Published:** 2022-02-03

**Authors:** Zhuang Xiong, Zhigang Dun, Yucheng Wang, Desheng Yang, Dongliang Xiong, Kehui Cui, Shaobing Peng, Jianliang Huang

**Affiliations:** National Key Laboratory of Crop Genetic Improvement, Ministry of Agriculture Key Laboratory of Crop Ecophysiology and Farming System in the Middle Reaches of the Yangtze River, College of Plant Science and Technology, Huazhong Agricultural University, Wuhan, China

**Keywords:** stomatal morphology, photosynthetic induction, stomatal kinetics, biochemical processes, intrinsic water use efficiency

## Abstract

Plants are often confronted with light fluctuations from seconds to minutes due to altering sun angles, mutual shading, and clouds under natural conditions, which causes a massive carbon loss and water waste. The effect of stomatal morphology on the response of leaf gas exchange to fluctuating light remains disputable. In this study, we investigated the differences in leaf stomatal morphology and photosynthetic induction across twelve rice genotypes after a stepwise increase in light intensity. A negative correlation was observed between stomatal size and density across rice genotypes. Smaller and denser stomata contributed to a faster stomatal response under fluctuating light. Plants with faster stomatal opening also showed faster photosynthetic induction and higher biomass accumulation but lower intrinsic water use efficiency (*_*i*_WUE*) under fluctuating light. Moreover, stomatal morphology seemed to have less effect on the initial and final stomatal conductance, and there was a minimal correlation between steady-state and non-steady-state stomatal conductance among different rice genotypes. These results highlight the important role of stomatal morphology in regulating photosynthetic efficiency and plant growth under fluctuating light conditions. To simultaneously enhance leaf *_*i*_WUE* when improving the photosynthetic efficiency under fluctuating light, it may be necessary to take biochemical processes into account in the future.

## Introduction

It is urgent to increase crop productivity to meet the demands of the growing population (Ashraf and Akram, 2009). Rice is one of the most important staple foods for more than half of the world’s population, especially throughout Asia, where nearly 90% of the population is dependent on it for most of their daily caloric intake (Dawe, 2000). Photosynthesis is widely accepted as a vital target to improve crop productivity due to its importance in supporting plant growth (Long et al., 2006; Lawson et al., 2012; Wu et al., 2019), although the direct relationship between leaf photosynthesis and the level of whole plant growth is still controversial. Moreover, plants are often confronted with light fluctuations due to altering sun angles, mutual shading, and clouds under natural conditions, which causes a massive carbon loss and water waste (Pearcy, 1988; Pearcy et al., 1990). To maximize carbon assimilation and water use, plants need to have a rapid photosynthetic response to fluctuating light (Qu et al., 2016). On the shift to illumination from a shading environment, the photosynthetic rate tends to exhibit a typical delay response before reaching a new steady-state, which takes tens of minutes and is called photosynthetic induction (Taylor and Long, 2017; Adachi et al., 2019). The photosynthetic induction is generally limited by three factors, including electron transport rate in the thylakoid membrane, activation of Calvin-Benson cycle enzymes, and stomatal movement (Pearcy et al., 1990). Comparatively speaking, the photosynthetic induction is mainly limited by stomatal kinetics, and only the biochemical process has a very short-term limiting effect due to the rapid activation rate of electron transport and Rubisco (Yamori et al., 2016; Deans et al., 2019; De Souza et al., 2020).

Stomatal kinetics is controlled by guard cell turgidity, which is sensitive to light intensity (Elliott-Kingston et al., 2016; Monda et al., 2016). Still, the underlying mechanisms for the stomatal movement under fluctuating light are not fully understood (Lawson and Vialet-Chabrand, 2019; Vialet-Chabrand et al., 2021). The stomatal morphology, including stomatal size, density, and spacing, has been widely accepted as the determinant of stomatal conductance (Franks and Beerling, 2009; Franks et al., 2009; Fanourakis et al., 2020). Also, many previous studies have demonstrated the general correlations between stomatal morphology and stomatal kinetics under fluctuating light, and a higher density of smaller stomata contributes to a faster stomatal response (Lawson and Blatt, 2014; Raven, 2014; Gerardin et al., 2018; Kardiman and Raebild, 2018). However, Elliott-Kingston et al. (2016) found that stomatal morphology is not correlated with the stomatal closing rate, as well as with the opening rate (McAusland et al., 2016). Zhang et al. (2019) suggested that larger size and lower density of stomata may promote the initial stomatal conductance at low light and decrease the stomatal delay during the initial phase after switching to high light conditions. Thus, the effect of stomatal morphology on stomatal kinetics and thereafter photosynthetic induction under fluctuating light remains to be further investigated.

Moreover, the maximum and minimum stomatal conductance is positively correlated with the maximum response rate of stomatal opening from low light to high light conditions (Drake et al., 2013). This is also supported by the findings of Auchincloss et al. (2014), who reported that pre-opened stomata at dawn could result in a faster response of daytime stomatal opening. However, Acevedo-Siaca et al. (2020a,b) suggested that there was no correlation between steady-state and non-steady-state photosynthetic rates. Further evidence for the relationship between steady-state and non-steady-state gas exchange is still needed. Plant intrinsic water use efficiency (*_*i*_WUE*) has always been an important issue with increasing demand to improve crop yield and the amount of carbon assimilation per unit of water used (Flexas, 2016). Previous studies have reported the important role of stomatal kinetics in *_*i*_WUE* under fluctuating light conditions, as stomatal kinetics is often a magnitude slower than photosynthetic response after a stepwise change in light intensity (Eyland et al., 2021). After switching from high light to low light, the faster response of stomatal closing can decrease the water loss and improve *_*i*_WUE* (Qu et al., 2020). As a matter of fact, a slower response of stomatal opening may be more likely to conserve water but will limit the photosynthetic response (Eyland et al., 2021). Thus, the target of simultaneously improving the photosynthetic efficiency and *_*i*_WUE* under fluctuating light conditions still deserves further exploration.

In this study, twelve rice genotypes were pot-grown in natural environments with sufficient nutrition. The variations of stomatal morphology and dynamic gas exchange across these genotypes were investigated. This study aimed to explore (1) the effect of stomatal morphology on the dynamic response of stomatal conductance and photosynthetic rate, (2) the relationship between the steady-state and non-steady-state gas exchange, and (3) the effect of stomatal kinetics on plant growth and *_*i*_WUE* under fluctuating light.

## Materials and Methods

Twelve rice genotypes, including T1 (4X), A1 (4X), WH (4X), Yangdao6 (4X), Yongyou12, Yangdao6 (2x), N22, WH (2x), Huanghuazhan (HHZ), Yangliangyou6 (YLY6), Guangzhan63 (GCA), and Guangchangai (GCA), were chosen in this study (Table 1). T1 (4X), A1 (4X), WH (4X), and Yangdao6 (4X) were tetraploid rice, among which WH (4X) and Yangdao6 (4X) were isogenic tetraploid of WH (2x) and YD6 (2x), respectively. After germination, the seeds were sown into nursery plates in the open air on February 5, 2018, in Hainan Province. Three seedlings per pot were transplanted into a 10-L plastic pot containing field paddy soil (wet) 20 days later. Eight pots were set per genotype in this study. About 3 g of nitrogen (N) per pot was applied in the form of urea, which was split-applied at a ratio of 4:3:3 at three phases including basal, tillering stage, and panicle initiation, and solid fertilizer was applied 7 days after transplanting. The application of phosphorus (P) and potassium (K) was 1.5 g per pot in the form of superphosphate and potassium chloride, respectively, which were mixed as the basal fertilizer. After transplanting, the plants were grown outdoors with natural irradiance and randomized on a weekly basis (Supplementary Figure 1). During the growing season, plants were well-watered, and a minimum of a 2-cm water layer was maintained in the pots.

**TABLE 1:** Information of genus *Oryza* used in this study.

Species	Ploidy	Genotypes	Abbreviation
/	Tetraploid	T1	T1
/	Tetraploid	A1	A1
/	Tetraploid	WH (4X)	WH (4X)
/	Tetraploid	Yangdao 6 (4X)	YD6 (4X)
Oryza sativa L.	Diploid	Yongyou 12	YY12
Oryza sativa L.	Diploid	Yangdao 6 (2X)	YD6 (2X)
Oryza sativa L.	Diploid	N22	N22
Oryza sativa L.	Diploid	WH (2X)	WH (2X)
Oryza sativa L.	Diploid	Huanghuazhan	HHZ
Oryza sativa L.	Diploid	Yangliangyou 6	YLY6
Oryza sativa L.	Diploid	Guangzhan 63	GZ63
Oryza sativa L.	Diploid	Guangchangai	GCA

### Measurements of Photosynthetic Induction

All gas exchange parameters were recorded using a Li-Cor 6400XT portable gas exchange system (Li-Cor, Lincoln, NE, United States) in the open air on sunny days. A 2 cm × 3 cm chamber was used, and a LED 6400-02B lamp served as the light source. Throughout the measurement, the reference infrared gas analyzer (IRGA) CO_2_ concentration was maintained at 400 μmol mol^–1^; the IRGA flow rate was set at 500 μmol s^–1^; the chamber temperature was kept at 28°C; and the leaf-to-air vapor-pressure deficit (VPD_*leaf*–*air*_) was maintained at 1.3 ± 0.2. All measurements were conducted using the youngest fully expanded leaf from 9:00 a.m. to 16:00 p.m. in April 2018.

**FIGURE 1:**
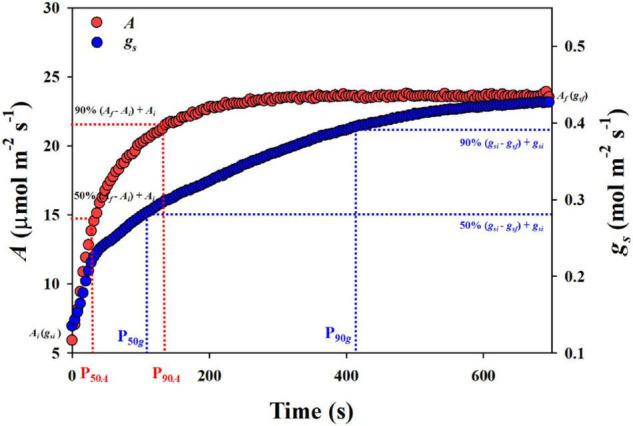
The calculation of stomatal conductance *g*_*s*_ and photosynthetic rate *A* in response to a stepwise increase in light intensity. P_50_*_*g*_* and P_90_*_*g*_*, the time taken for *g*_*s*_ to reach 50 and 90% of the difference between the initial and final *g*_*s*_. P_50_*_*A*_* and P_90_*_*A*_*, the time taken for *A* to reach 50 and 90% of the difference between the initial and final *A*.

During the measurements, the leaf was first equilibrated at a photosynthetically active photon flux density (PPFD) of 100 μmol m^–2^ s^–1^ until the photosynthetic rate (*A*) and stomatal conductance (*g*_*s*_) reached a steady state. Then, the PPFD was increased to 1,500 μmol m^–2^ s^–1^ until 600 s of light induction for *A* and *g*_*s*_. The data were recorded every 4 s. To estimate the response of stomatal opening to a stepwise increase in light intensity, P_50_*_*g*_* and P_90_*_*g*_* were calculated, which represent the time required for *g*_*s*_ to reach 50 and 90% of the difference between the initial and final stomatal conductance (*g*_*si*_ and *g*_*sf*_) after switching to high light conditions. Similarly, the response rate of photosynthesis to a stepwise increase in light intensity was obtained by calculating the time required for *A* to reach 50 and 90% (P_50_*_*A*_* and P_90_*_*A*_*) of the difference between the initial and final photosynthetic rate (*A*_*i*_ and *A*_*f*_) (Figure 1).

Carbon gain (*C*_*g*_) and Carbon loss (*C*_*l*_) represent the integrated amount of CO_2_ uptake and loss during light induction, which were calculated as follows:


$$Carbongain=A_{t}^{*}d_{t}$$

$$Carbonloss=A_{f}^{*}△{\text{t-A}}_{t}^{*}d_{t}$$

where *A*_*t*_ represents the transient photosynthetic rate during light induction, and *A*_*f*_ is the final rate at the end of light induction.

### Stomatal Morphology

The epidermal impressions were collected from the abaxial surface of the youngest fully expanded leaves. The middle segment of leaf samples was fixed in formalin-acetic acid-alcohol (FAA) solution. The abaxial surface of the leaf was obtained by gently scratching the adaxial surface using blades. Afterward, the transparent epidermis was kept in clean water. The epidermal impressions were placed on the microscope slides and analyzed using an optical microscope equipped with a digital camera. In each treatment, five slides from three plants were used for determination. Stomatal density and number were determined using ImageJ. Five stomata were randomly selected from each slide to determine the stomatal size, which was calculated by multiplying stomatal length and width.

### Plant Growth

Once all gas exchange and stomatal morphology measurements were accomplished, one plant was sampled per pot to estimate the plant growth. The plants were then separated into leaves and stems. A leaf area meter (LI-3000, LI-COR Inc., Lincoln, NE, United States) was used to determine the total leaf area. Besides, the number of stems per plant was recorded. Finally, to determine the total dry weight of the aboveground part, leaves and stems were oven-dried at 80°C to constant weight.

### Statistical Analysis

One-way ANOVA and the least-significant difference (LSD) test were used to assess the measured parameters using SPSS 21.0 (SPSS for Windows, Chicago, IL, United States). Linear regression was analyzed to test the correlations between the measured parameters using SigmaPlot 12.5 (Systat Software Inc., San Jose, CA, United States).

## Results

### Variations of Gas Exchange Across Rice Genotypes

**FIGURE 2:**
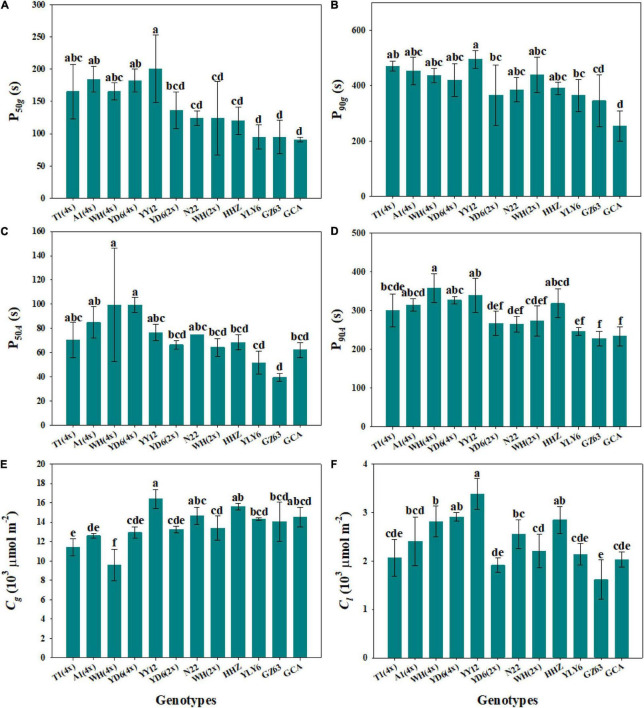
Response rate of gas exchange parameters under a stepwise increase in light intensity across 12 rice genotypes. **(A,B)** Time taken for *g*_*s*_ to reach 50% (P_50_*_*g*_*) and 90% (P_90_*_*g*_*) of the difference between the initial and final values. **(C,D)** Time taken for *A* to reach 50% (P_50_*_*A*_*) and 90% (P_90_*_*A*_*) of the difference between the initial and final values. **(E,F)** Integrated amount of carbon gain (*C*_*g*_) and carbon loss (*C*_*l*_) during 600 s of photosynthetic induction. Each bar represents the mean ( ± SD) of three replications. Different letters indicate statistically significant differences (*P* < 0.05) among twelve rice genotypes.

**FIGURE 3:**
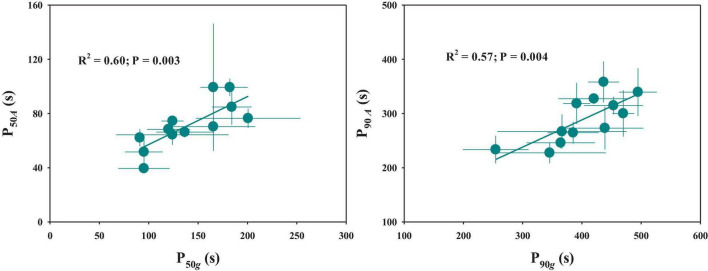
Relationship between stomatal kinetics and photosynthetic induction. P_50_*_*g*_* and P_50_*_*A*_* represent the time taken for *g*_*s*_ and *A* to reach 50% of the difference between the initial and final values, respectively. P_90_*_*g*_* and P_90_*_*A*_* represent the time taken for *g*_*s*_ and *A* to reach 90% of the difference between the initial and final values, respectively. Points and error bars represent mean ± SD of three replications.

The response of stomatal conductance to a stepwise increase in light intensity was determined by calculating the P_50_*_*g*_* and P_90_*_*g*_* among twelve rice genotypes (Figure 1). P_50_*_*g*_* and P_90_*_*g*_* showed significant variations among rice genotypes, ranging from 91 to 200.7 s and 254.5 to 469.8 s, respectively (Figures 2A,B). To estimate the effect of stomatal kinetics on the photosynthetic rate under fluctuating light, we also calculated P_50_*_*A*_* and P_90_*_*A*_*. Similarly, significant variations were observed among different rice genotypes in P_50_*_*A*_* and P_90_*_*A*_*, which ranged from 39.5 to 99.3 s and from 227.5 to 358.2 s, respectively (Figures 2C,D). Interestingly, tetraploid rice showed a significantly slower response of stomatal conductance and photosynthetic rate to fluctuating light than diploid rice, since P_90_*_*g*_* and P_90_*_*A*_* were higher for WH (4x) and YD6 (4x) than for WH (2x), and YD6 (2x), and it was the same case for P_50_*_*g*_* and P_50_*_*A*_*. Leaf carbon gain (*C*_*g*_) and carbon loss (*C*_*l*_) significantly varied among twelve rice genotypes during 600 s of light induction (Figures 2E,F). A significant positive correlation was observed between P_50_*_*g*_* and P_50_*_*A*_*, as well as between P_90_*_*g*_* and P_90_*_*A*_* (Figure 3).

**FIGURE 4:**
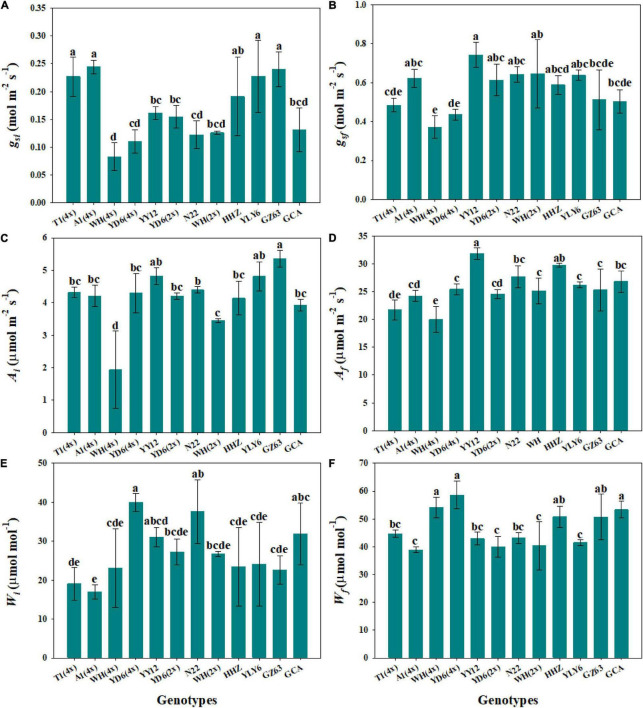
Steady-state gas exchange parameters. **(A,B)** Initial and final stomatal conductance (*g*_*si*_ and *g*_*sf*_). **(C,D)** Initial and final photosynthetic rate (*A*_*i*_ and *A*_*f*_). **(E,F)** Initial and final water use efficiency (*W*_*i*_ and *W*_*f*_) during light induction. Each bar represents the mean ( ± SD) of three replications. Different letters indicate statistically significant differences (*P* < 0.05) among twelve rice genotypes.

**FIGURE 5:**
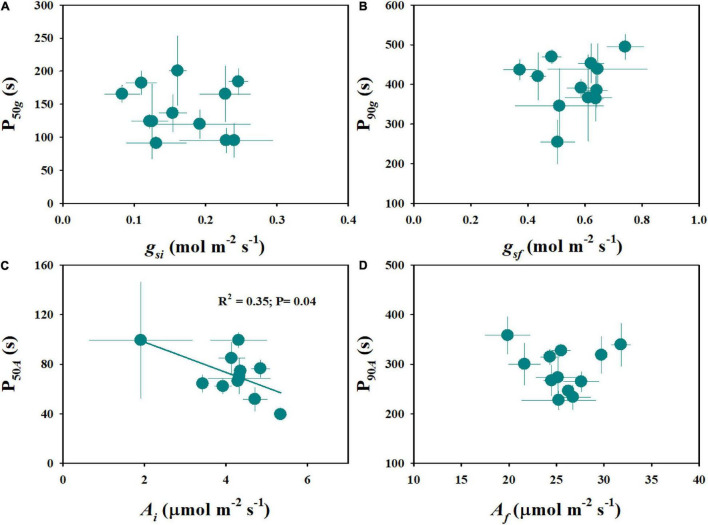
Relationship between steady-state and non-steady-state gas exchange. **(A,B)** Initial stomatal conductance (*g*_*si*_) and initial response rate of stomatal conductance (P_50_*_*g*_*), final stomatal conductance (*g*_*sf*_), and final response rate of stomatal conductance (P_90_*_*g*_*). **(C,D)** Initial photosynthetic rate (*A*_*i*_) and initial response rate of photosynthetic rate (P_50_*_*A*_*), final photosynthetic rate (*A*_*f*_), and final response rate of photosynthetic rate (P_90_*_*A*_*). Points and error bars represent mean ± SD of three replications.

Steady-state gas exchange is considered as an important factor affecting the dynamic response to fluctuating light. We observed significant differences in initial and final stomatal conductance (*g*_*si*_ and *g*_*sf*_) and photosynthetic rate (*A*_*i*_ and *A*_*f*_) among rice genotypes (Figures 4A–D and Supplementary Figure 2). Moreover, the tetraploid rice of WH (4x) and YD6 (4x) showed significantly lower *g*_*sf*_ and *A*_*f*_ than WH (2x) and YD6 (2x) (Figures 4B,D). The initial and final water use efficiency (*W*_*i*_ and *W*_*f*_) also significantly varied among rice genotypes (Figures 4E,F). Significantly lower *W*_*i*_ and *W*_*f*_ were observed in diploid rice than in tetraploid rice. No significant correlation was observed between *g*_*si*_ and P_50_*_*g*_*, as well as between *g*_*sf*_ and P_90_*_*g*_* (Figures 5A,B). However, *A*_*i*_ was negatively correlated with P_50_*_*A*_*, and no correlation between *A*_*f*_ and P_90_*_*A*_* was observed (Figures 5C,D). Leaf *_*i*_WUE* was mainly determined by stomatal conductance under fluctuating light (Supplementary Figure 3).

### Relationship Between Stomatal Morphology and Stomatal Kinetics

**FIGURE 6:**
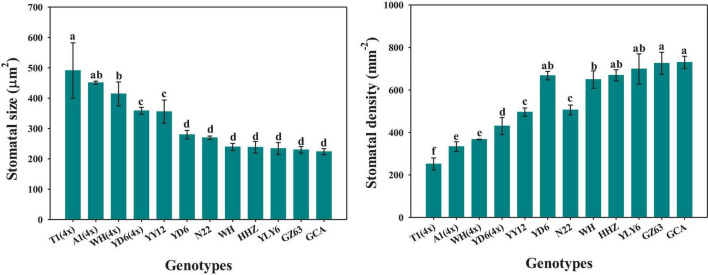
Variations of stomatal size and density across twelve rice genotypes. Different letters indicate statistically significant differences (*P* < 0.05) among twelve rice genotypes.

**FIGURE 7:**
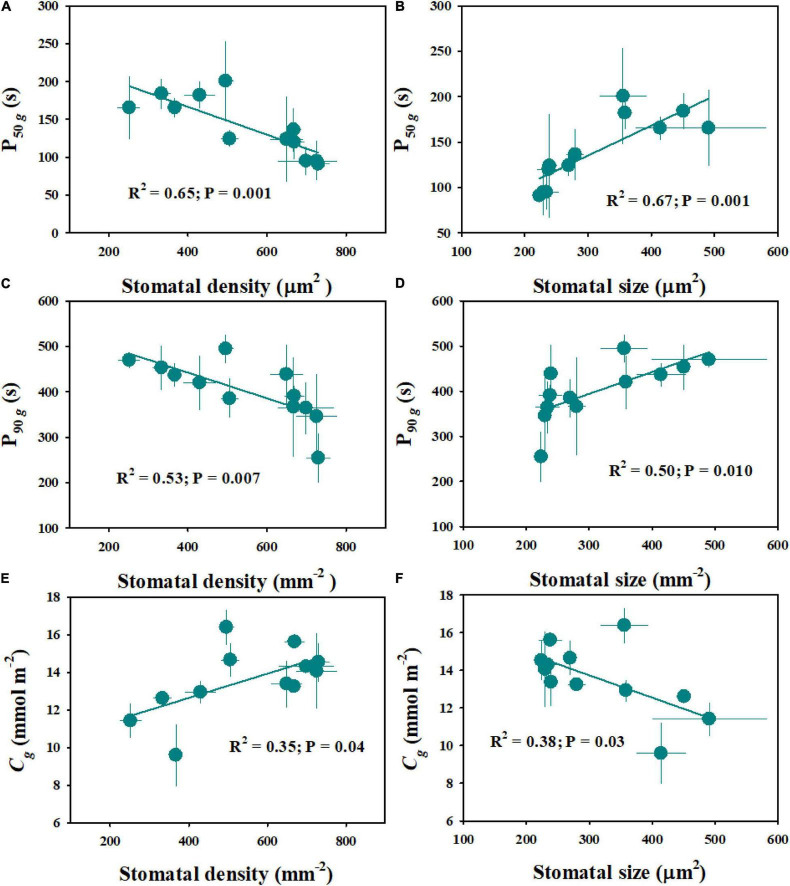
Effect of stomatal morphology (stomatal density and stomatal size) on stomatal kinetics **(A–D)** and carbon gain **(E,F)**. Stomatal kinetics (P_50_*_*g*_*, P_90_*_*g*_*), the time required for *g*_*s*_ to reach 50 and 90% of the difference in *g*_*si*_ and *g*_*sf*_; *C*_*g*_, integrated amount of CO_2_ uptake. Points and error bars represent the mean ± SD of three replications.

The rice genotypes varied significantly in stomatal size and density, ranging from 224 to 491 μm^2^ and from 252 to 730 mm^–2^, respectively (Figure 6). Moreover, significant differences were observed in stomatal size and density between diploid and tetraploid rice (Figure 6). Compared with WH (2x) and YD6 (2x), WH (4x) and YD6 (4x) exhibited significantly larger stomatal size and lower stomatal density. The stomatal density was found to have significant negative correlations with both P_50_*_*g*_* and P_90_*_*g*_* (Figures 7A,C). Inversely, the stomatal size was significantly positively correlated with both P_50_*_*g*_* and P_90_*_*g*_* (Figures 7B,D). Also, a higher density of smaller stomata could contribute to a faster photosynthetic induction and higher carbon gain (*C*_*g*_) (Figures 7E,F).

### Effect of Photosynthetic Induction on Plant Growth Across Rice Genotypes

**FIGURE 8:**
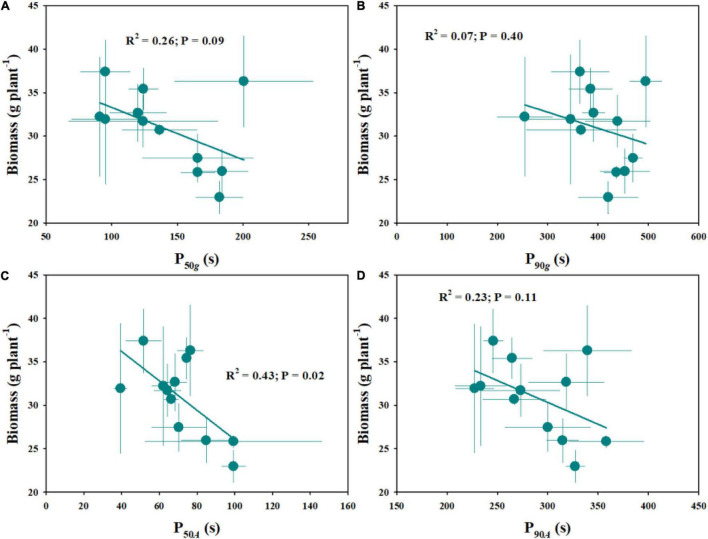
Effect of stomatal kinetics **(A,B)** and photosynthetic induction **(C,D)** on plant biomass. Stomatal kinetics (P_50_*_*g*_*, P_90_*_*g*_*), the time required for *g*_*s*_ to reach 50 and 90% of the difference in *g*_*si*_ and *g*_*sf*_; photosynthetic induction (P_50_*_*A*_*, P_90_*_*A*_*), the time required for *g*_*s*_ to reach 50 and 90% of the difference in *A*_*i*_ and *A*_*f*_. Points and error bars represent the mean ± SD of three replications.

The natural variations of plant growth were further explored within twelve rice genotypes, including the number of tillers, total leaf area, leaf mass per area (LMA), and biomass (Table 2). Similarly, significant variations of the abovementioned indices were observed across different rice genotypes. Moreover, compared with diploid rice, tetraploid rice showed significantly fewer tillers, a smaller total leaf area and, therefore, lower biomass. The stomatal kinetics and photosynthetic induction showed negative correlations with the plant biomass (Figure 8).

**TABLE 2:** Differences of plant growth across twelve rice genotypes.

Species	Tillers (No.)	Total leaf area (10^3^ cm^2^ Plant^–1^)	LMA (10^–3^ g/cm^2^)	Biomass (g Plant^–1^)
T1 (4X)	13.0 ± 1.0 d	1.40 ± 0.17 ef	5.30 ± 0.36 b	27.5 ± 2.8 bc
A1 (4X)	13.3 ± 0.6 d	1.42 ± 0.04 def	5.4 ± 80.14 b	26.0 ± 2.6 bc
WH (4X)	12.3 ± 0.6 d	1.27 ± 0.03 fg	5.59 ± 0.21 b	25.8 ± 0.7 bc
YD6 (4X)	12.3 ± 0.6 d	1.06 ± 0.07 g	6.63 ± 0.12 a	23.0 ± 1.9 c
YY12	17.0 ± 1.0 c	1.45 ± 0.11 def	4.55 ± 0.17 c	36.3 ± 5.2 a
YD6 (2x)	17.7 ± 1.5 c	1.64 ± 0.04 cde	5.76 ± 0.01 b	30.7 ± 0.6 ab
N22	24.3 ± 2.5 a	1.80 ± 0.16 abc	4.25 ± 0.08 c	35.4 ± 2.4 a
WH (2x)	16.7 ± 1.5 c	1.98 ± 0.15 ab	4.44 ± 0.15 c	31.7 ± 3.0 ab
HHZ	21.7 ± 1.2 b	1.71 ± 0.21 bcd	5.49 ± 0.45 b	32.7 ± 3.3 ab
YLY6	18.7 ± 1.2 c	2.00 ± 0.28 a	5.43 ± 0.13 b	37.4 ± 3.7 a
GZ63	17.7 ± 2.1 c	1.60 ± 0.24 cde	5.51 ± 0.37 b	31.9 ± 7.5 ab
GCA	18.7 ± 1.5 c	1.54 ± 0.11 adef	5.62 ± 0.37 b	32.2 ± 6.9 ab

*All data are mean ± SD of three replications. Different letters indicate statistically significant differences (P < 0.05) among twelve rice genotypes.*

## Discussion

### Smaller and Denser Stomata Contribute to Faster Photosynthetic Induction

Stomatal conductance (*g*_*s*_) is known to be determined by stomatal morphology and aperture. Generally, there are significant negative correlations between stomatal size and density across or within species (Franks et al., 2009; Fanourakis et al., 2015), which is also supported by our results (Figure 6). Numerous studies have reported that stomatal morphology has strong correlations with the *g*_*s*_ and photosynthetic rate (*A*) under constant light conditions (Franks and Beerling, 2009; Xiong et al., 2017; Xiong et al., 2018). Recently, some studies have reported that stomatal movement in response to environmental fluctuations is often affected by stomatal morphology (Lawson et al., 2014; Fanourakis et al., 2020); however, other studies have suggested that there is no correlation between stomatal kinetics and stomatal morphology (Eyland et al., 2021). In this study, significant variations were observed in stomatal morphology and stomatal response rate to fluctuating light among different rice genotypes (Figures 2A,B, 6). Interestingly, the rate of stomatal response to fluctuating light was significantly positively correlated with the stomatal size, while negatively correlated with the stomatal density (Figures 7A,B), which is in line with the findings of Drake et al. (2013). It is worth noting that P_50_*_*g*_* has a stronger correlation with the stomatal morphology than P_90_*_*g*_*, suggesting that the initial phase of stomatal response might be more likely affected by stomatal morphology (Figures 7A–D). Moreover, the tetraploid rice WH (4x) and YD6 (4x) showed a larger size and lower density of stomata and correspondingly slower stomatal response than WH (2x) and YD6 (2x), which again indicates that stomatal morphology plays an important role in regulating stomatal kinetics under fluctuating light (Figures 2A,B, 6).

Many studies have been focused on the coordination between *g*_*s*_ and *A* under fluctuating light conditions (Adachi et al., 2019; Kimura et al., 2020; Sakoda et al., 2021). After shifting to illumination from a shading environment, stomatal opening often shows a typical delay response relative to photosynthetic induction, which will result in a stomatal limitation to *A* (McAusland et al., 2016). Several studies have investigated the key limiting factors during photosynthetic induction, among which *g*_*s*_ is the main factor that limits *A* during light induction, and the biochemical processes only have a very short-term limiting effect at the initial phase (Kaiser et al., 2016; Adachi et al., 2019; De Souza et al., 2020; Eyland et al., 2021). However, Acevedo-Siaca et al. (2020a,b) proposed that photosynthesis is primarily limited by biochemistry, especially the activation of RuBisCo under fluctuating light. In this study, we observed significant differences in photosynthetic induction across twelve rice genotypes (Figures 2C,D). Also, we found a significant contribution of stomatal opening to photosynthetic response under a stepwise increase in light intensity, since P_50_*_*g*_* and P_90_*_*g*_* were positively correlated with P_50_*_*A*_* and P_90_*_*A*_*, respectively (Figure 3). Therefore, a higher density of smaller stomata may contribute to faster stomatal kinetics and photosynthetic induction under fluctuating light.

### Steady-State and Non-steady-State Gas Exchange Are Not Correlated With Each Other

Many studies have been focused on the underlying mechanisms of light-induced stomatal movement, which may be triggered by the products of the photosynthetic process in guard cells or mesophyll cells, but the exact signals remain unclear (Lawson, 2009; Lawson et al., 2014; Santelia and Lawson, 2016). Drake et al. (2013) and Zhang et al. (2019) reported that higher initial and final *g*_*s*_ contribute to a faster *g*_*s*_ response to fluctuating light. A higher initial *g*_*s*_ at low light may reduce the initial lag (λ) and promote the initial response of stomatal conductance and photosynthetic rate to fluctuating light (Adachi et al., 2019). Differently, in this study, *g*_*si*_ and *g*_*sf*_ showed no correlation with P_50_*_*g*_* and P_90_*_*g*_*, respectively (Figures 5A,B). It has been widely accepted that stomatal morphology determines the potential maximum *g*_*s*_ under a steady state (Franks and Beerling, 2009; Franks et al., 2009). However, stomatal morphology showed no significant effect on *g*_*sf*_ in this study (Supplementary Figure 4), possibly because the measurement of *g*_*sf*_ during light induction cannot accurately reflect the potential maximum *g*_*s*_.

Light is one of the most dynamic factors under natural conditions, which often results in fluctuations of gaseous exchange on the leaf surface (Durand et al., 2019). Notably, the steady-state measurement generally cannot accurately indicate leaf photosynthetic efficiency in the natural environment when considering leaf carbon uptake. Currently, several studies have reported the low correlation between steady-state and non-steady-state photosynthesis (Acevedo-Siaca et al., 2020a,b). Consistently, less correlation was observed between steady-state and non-steady-state photosynthesis in this study (Figures 5C,D). One possible reason is the trade-off between photosynthetic proteins inside leaves, including RuBisCo and RuBisCo activase content, which determines the difference between steady-state and non-steady-state photosynthesis (Acevedo-Siaca et al., 2020b). Thus, further evidence is still needed to evaluate the relationship between the steady-state and non-steady-state gaseous exchange, which will facilitate the improvement of leaf photosynthetic efficiency under natural conditions in the future.

### Stomatal Kinetics Affects Plant Biomass and Water Use Efficiency

The improvement of photosynthesis has always been a major target to increase crop yield to meet the demand of the increasing global population. However, the relationship between leaf photosynthesis and plant growth is not always predictable, since there are various confounding factors arising from plant growth, developmental dynamics, and complex growing environments (Wu et al., 2019). Fluctuating light is a common factor affecting plant carbon uptake under natural conditions (Durand et al., 2019). In this study, we estimated the differences in leaf gas exchange in response to fluctuating light across twelve rice genotypes and the influence on plant biomass. As a result, stomatal kinetics and photosynthetic induction showed negative correlations with the plant biomass (Figure 8). Faster stomatal kinetics contributes to a higher photosynthetic rate under fluctuating light and, thereafter, higher biomass accumulation, which is in line with the results reported by Kimura et al. (2020). These results again suggest that stomatal morphology plays an important role in regulating leaf photosynthetic induction and plant biomass accumulation under dynamic environmental conditions.

Stomata are micropores composed of pairs of guard cells on the epidermis of leaves, which control the balance of CO_2_ uptake for photosynthesis and water loss *via* transpiration. Low *g*_*s*_ to water vapor can conserve water by limiting CO_2_ uptake for *A*. With a stepwise increase in light intensity, the *g*_*s*_ and *A* displayed asynchronous responses, as stomatal kinetics are often a magnitude slower than photosynthetic induction, which will result in at least a 20% decrease in *_*i*_WUE* (Lawson et al., 2014). This asynchronous response was also observed in this study. During the initial phase of induction, photosynthesis was mainly limited by biochemistry, and the *g*_*s*_ was higher than needed, resulting in a higher *C*_*i*_ and lower *_*i*_WUE* (Supplementary Figures 2C,D). Also, we observed a significant negative correlation between steady-state water use efficiency (*W*_*i*_ and *W*_*f*_) and stomatal conductance (*g*_*si*_ and *g*_*sf*_), which again indicates that excessively higher *g*_*s*_ will decrease the *_*i*_WUE* (Supplementary Figures 3A,C).

## Conclusion

This study also shows that genotypes with larger stomatal sizes generally have a lower stomatal density. A higher density of smaller stomata will contribute to faster stomatal kinetics and, thereafter, higher biomass accumulation but reduce the leaf *_*i*_WUE*. Further evidence is still needed to evaluate the relationship between steady-state and non-steady-state gas exchange. This study mainly highlights the important role of stomatal morphology in regulating leaf photosynthetic induction and plant growth. To simultaneously improve photosynthetic efficiency and *_*i*_WUE*, it may be necessary to take biochemical processes into account in the future.

## Data Availability Statement

The raw data supporting the conclusions of this article will be made available by the authors, without undue reservation.

## Author Contributions

ZX and JH planned and designed the experiment and analyzed the data and wrote the manuscript. ZX and ZD performed the plant propagation and leaf gas exchange experiments. YW and DY performed the stomatal anatomy experiment. All authors revised the manuscript.

## Conflict of Interest

The authors declare that the research was conducted in the absence of any commercial or financial relationships that could be construed as a potential conflict of interest.

## Publisher’s Note

All claims expressed in this article are solely those of the authors and do not necessarily represent those of their affiliated organizations, or those of the publisher, the editors and the reviewers. Any product that may be evaluated in this article, or claim that may be made by its manufacturer, is not guaranteed or endorsed by the publisher.
