# Supplementary material for: Effect of Stomatal Morphology on Leaf Photosynthetic Induction Under Fluctuating Light in Rice
Source: Front Plant Sci. 2022 Feb 3;12:754790. doi: 10.3389/fpls.2021.754790 (PMC8851391; doi:10.3389/fpls.2021.754790)
Supplement: Supplementary file 1 [file Image_1.pdf]

Effects of stomatal morphology on leaf photosynthetic induction under fluctuating light in rice

Zhuang Xiong, Zhigang Dun, Yucheng Wang, Desheng Yang, Dongliang Xiong, Kehui Cui, Shaobing Peng, Jianliang Huang*

[1] National Key Laboratory of Crop Genetic Improvement, Ministry of Agriculture Key Laboratory of Crop Ecophysiology and Farming System in the Middle Reaches of the Yangtze River, College of Plant Science and Technology, Huazhong Agricultural University, Wuhan, Hubei 430070, China.

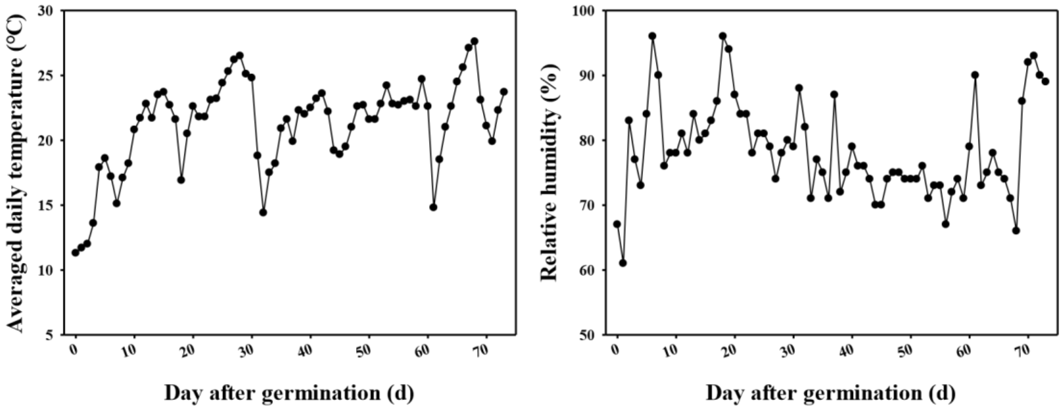

Figure S1 Average daily temperature (°C) and relative humidity (%) in the whole growing season. Each point represents the mean of daily temperature or relative humidity after germination.

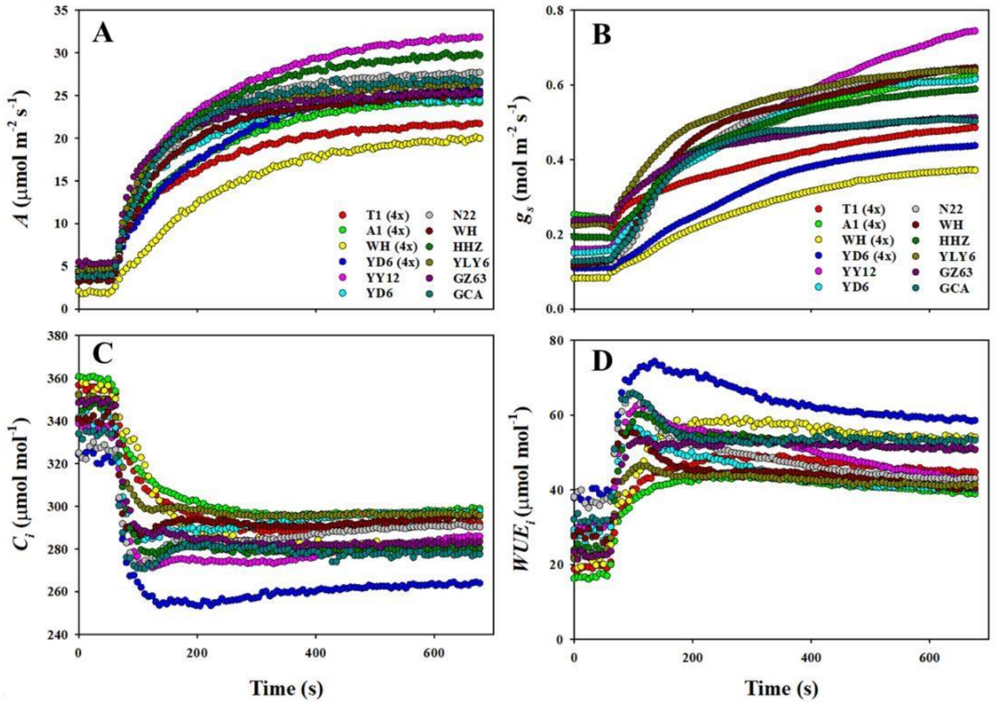

Figure S2 Response of gas exchange to a stepwise increase of light intensity among twelve rice genotypes. (A) Net CO_2 assimilation (*A*). (B) Stomatal conductance (g_s). (C) Intercellular CO_2 concentration (C_i). (D) Intrinsic water use efficiency (WUE_i). Each point represent mean of three replications.

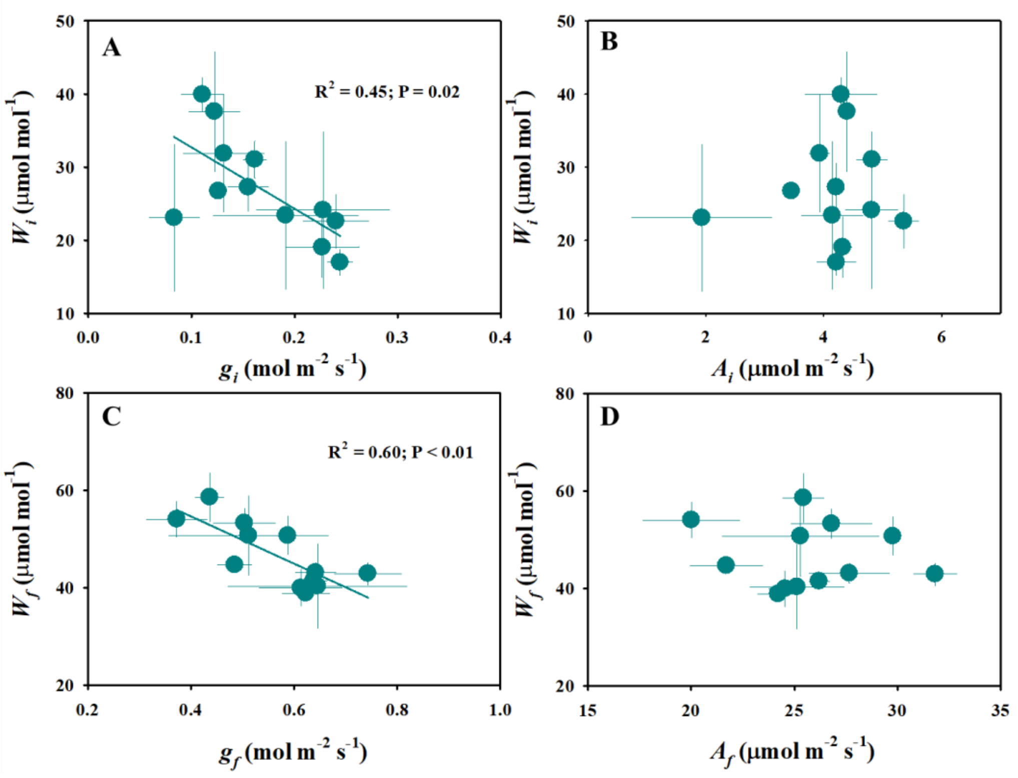

Figure S3 Relationship between stomatal conductance (g_{si}, g_{sf}) and intrinsic water use efficiency (A, C), as well as photosynthetic rate (A_i, A_f) and intrinsic water use efficiency (B, D) in the initial and final phase of light induction. g_{si} and g_{sf}, initial and final stomatal conductance; A_i and A_f, initial and final photosynthetic rate; W_i and W_f, initial and final water use efficiency. Points and error bars represent mean ± SD of three replications.

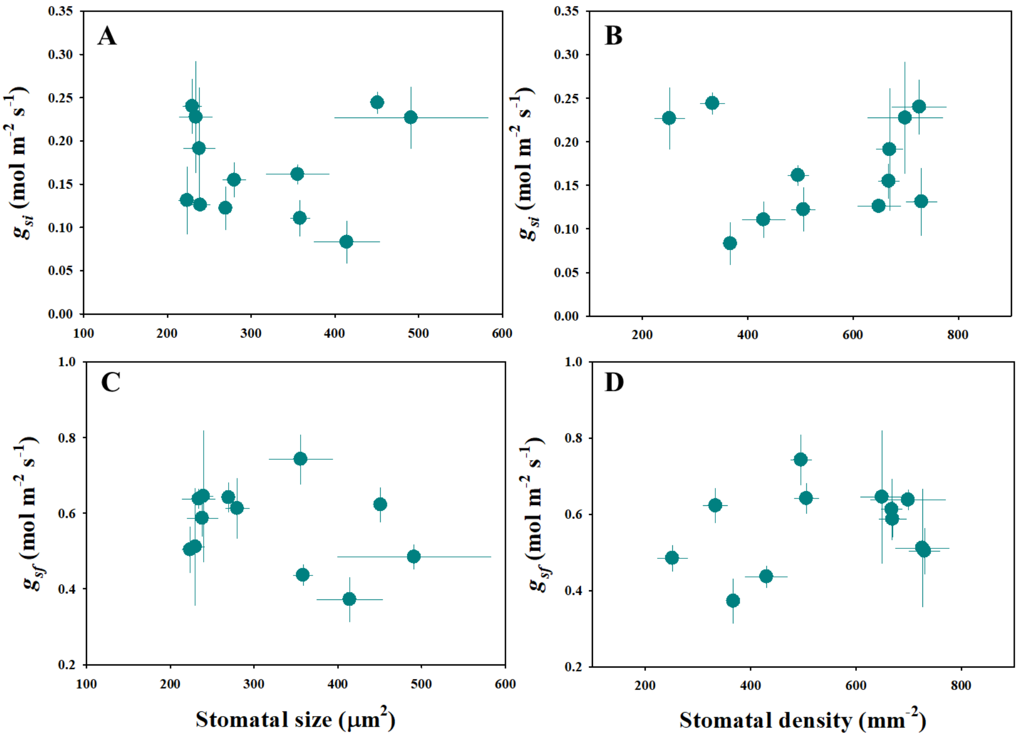

Figure S4 Effect of stomatal morphology on initial and final stomatal conductance during light-induction (A-D). g_{si} and g_{sf} represent initial and final stomatal conductance, respectively. Points and error bars represent mean ± SD of three replications.
